# Nucleolin mediates the antiangiogenesis effect of the pseudopeptide N6L

**DOI:** 10.1186/1471-2121-13-32

**Published:** 2012-11-13

**Authors:** Charalampos Birmpas, Jean Paul Briand, Josẻ Courty, Panagiotis Katsoris

**Affiliations:** 1 Department of Biology, University of Patras, Patras, Greece; 2 Université Paris Est Créteil, CNRS, Créteil Cedex, France; 3 CNRS, Institut de Biologie Moléculaire et Cellulaire, Strasbourg, France

**Keywords:** Angiogenesis, Nucleolin, Cancer, N6L, HB-19

## Abstract

**Background:**

Nucleolin is a protein over-expressed on the surface of activated cells. Recent studies have underlined the involvement of cell surface nucleolin in angiogenesis processes. This cell surface molecule serves as a receptor for various ligands implicated in pathophysiological processes such as growth factors, cell adhesion molecules like integrins, selectins or laminin-1, lipoproteins and viruses. N6L is a synthetic multimeric pseudopeptide that binds cell surface expressed nucleolin and inhibits cell proliferation.

**Results:**

In the present work, we further investigated the mechanisms of action of pseudopeptide N6L on angiogenesis using HUVECs. We provide evidence that N6L inhibits the in vitro adhesion, proliferation and migration of HUVECs without inducing their apoptosis. In addition, we found that N6L downregulates MMP-2 in HUVECs. The above biological actions are regulated by SRC, ERK1/2, AKT and FAK kinases as we found that N6L inhibits their activation in HUVECs. Finally, down regulation of nucleolin using siRNA demonstrated the implication of nucleolin in the biological actions of these peptides.

**Conclusions:**

Taken together, these results indicate that N6L could constitute an interesting therapeutic tool for treating diseases associated with excessive angiogenesis.

## Background

A multifunctional protein, nucleolin is ubiquitously expressed in exponentially growing eukaryotic cells. It was first described in 1973 as a protein involved in ribosome biogenesis and also in DNA and RNA metabolism [1]. More recently, nucleolin was shown to shuttle between cytoplasm and cell surface. In the cytoplasm, it provides a post-transcriptional regulation of strategic mRNA, participates in rRNA maturation and ribosome assembly and is involved in nucleo-cytoplasmic transport [1,2]. On the cell surface, it serves as a low affinity receptor for several ligands such as certain growth factors [3].

Following the first report of surface expression in hepatocarcinoma cells [4], more data have been added for the enhanced expression of nucleolin on the surface of tumor and endothelial cells, and in endothelial cells of the tumor vasculature [3-5] in which its expression is constantly induced [6]. These data, point out the involvement of cell-surface expressed nucleolin in cell proliferation in tumor cell growth but also in activated endothelial cells. The expression of nucleolin is enhanced on the surface of endothelial cells upon stimulation with the vascular endothelial growth factor (VEGF), and the functional blockade or down regulation of surface nucleolin in endothelial cells inhibits the migration of endothelial cells and prevents capillary-tubule formation [7].

Several reports have pointed out molecules related to cell proliferation or cell differentiation as ligands for cell surface nucleolin. Among these molecules are the hepatocyte growth factor, the heparin affin regulatory peptide, midkine, epithelial growth factor receptor ErbB and endostatin, which all play a significant role in tumor development and angiogenesis processes [8-13]. In addition several molecules like laminin-1, factor J, L- and P-selectins which regulate cell adhesion, leukocyte trafficking, inflammation and angiogenesis are also surface nucleolin binding proteins [14-17]. Furthermore, urokinase which is involved in mechanisms regulating pericellular proteolysis, cell-surface adhesion and mitogenesis, binds and is co-internalized with surface nucleolin [18,19].

In a previous study, we reported that the nucleolin binding multivalent pseudopeptide N6L suppressed both tumor growth and angiogenesis [20]. In vitro, N6L reduces tumor cell growth in soft agar assay in several carcinoma cell lines and the proliferation of endothelial cells. These activities in both tumor and activated endothelial cells lead to tumor growth inhibition in breast carcinoma MDA-MB 231 xenografts in athymic nude mice without displaying any toxicity in normal tissues [20]. Finally, N6L promotes tumor cell death in vitro and in vivo experiments [20].

In this study, we have investigated the anti-angiogenic activities and the mechanism of action of N6L on human umbilical vein endothelial cells (HUVEC) and the role of nucleolin in these activities.

## Results

### N6L inhibits adhesion, proliferation and migration of HUVECs

**Figure 1:**
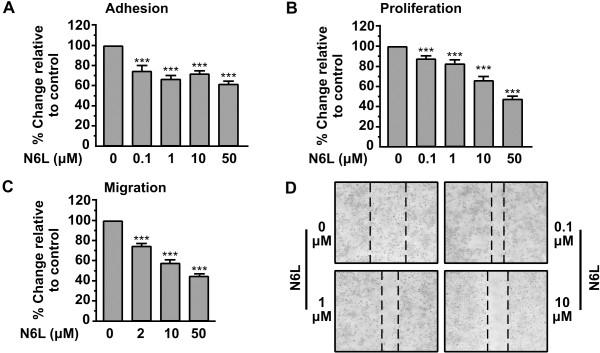
**N6L inhibits the in vitro adhesion, proliferation, migration and motility of HUVECs. (A)** Inhibition of HUVEC cell adhesion by N6L. An equal number of HUVECs were incubated with increasing concentrations of N6L for 30 min before seeding. After a 45 min incubation period, adherent cells were measured by the crystal violet assay. Results are expressed as % change relative to control and are mean values ± SE from at least 3 independent experiments **(B)**. Inhibition of HUVECs proliferation by N6L. Cells were cultured for 3 days in the presence of increasing concentrations of N6L. Cell proliferation was quantified by crystal violet staining. Results are expressed as % change relative to control and are mean values ± SE from at least 3 independent experiments. **(C)** Migration of cells through Transwell filters. The lower compartment of Transwell filters (8 μm pores) was filled with growth media containing 0.5% BSA, and increasing concentrations of N6L. An equal number of HUVECs was resuspended in a growth medium containing 0.5% BSA, and transferred into Transwell inserts. Cells that successfully migrated through the filter pores, were fixed, stained and quantified by counting the entire area of each filter. Results are expressed as % change relative to control and are mean values ± SE from at least 3 independent experiments. **(D)** Confluent cell monolayers were scratched and cells were left to heal the wound in the presence of increasing concentrations of N6L. 48 h later the plates were photographed. The dashed lines indicate the remaining wound.

The effect of N6L on the adhesion of HUVECs was first investigated. As shown in Figure 1A, N6L significantly inhibited the adhesion in a concentration-dependent manner, reaching a maximal effect at a concentration of 50 μΜ yielding 40% inhibition compared to the control (Figure 1A). All cells will adhere if left for more than 6 h so we investigated the effect of N6L on the proliferation of HUVECs. As shown in Figure 1B, N6L inhibited cell proliferation in a concentration dependent manner, having a maximal effect at a concentration of 50 μΜ (Figure 1B). The effect of N6L on HUVEC chemotaxis indicated that N6L inhibited chemotactic migration in a concentration-dependent manner with a maximal effect (61% inhibition relative to control) observed at the concentration of 50 μM (Figure 1C). Furthermore, the effect of N6L has been studied in wound-closure assays. As shown in Figure 1D, N6L used at a concentration of 10 μM completely inhibits HUVEC motility.

**Figure 2:**
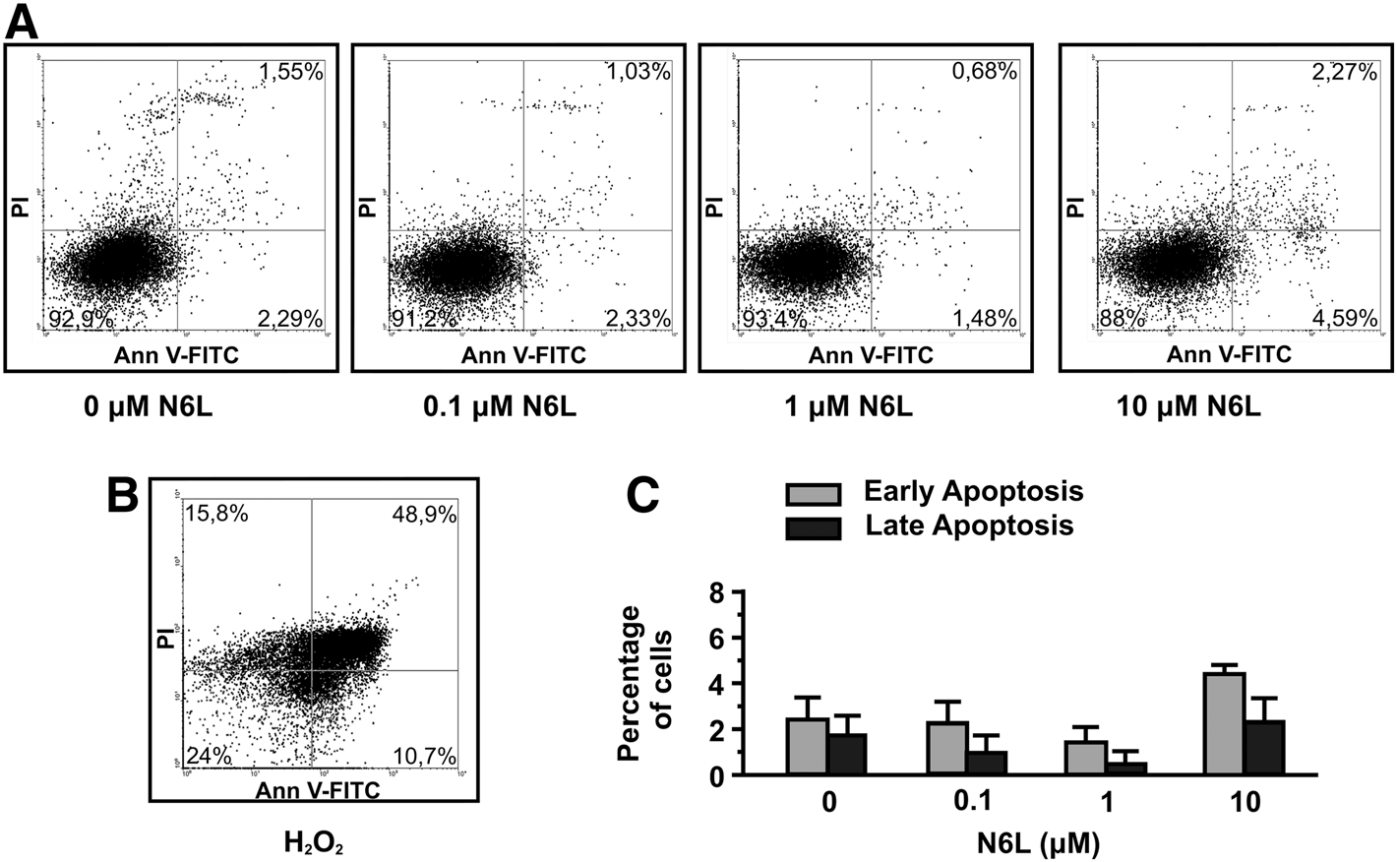
**N6L does not induce apoptosis of HUVECs.** Endothelial cells were incubated with increasing concentrations of N6L and 24 h later the number of apoptotic cells was measured by FACS analysis (**A** and **C**). Endothelial cells cultured in the presence of H_2_O_2 _were used as positive control (**B**).

To confirm that N6L has a direct effect on adhesion and migration and secondarily on proliferation without affecting cell survival, a cytotoxic assay using HUVEC, treated or not with various concentrations of N6L, has been performed. As shown in Figure 2, treatment of HUVEC with N6L for 24 h did not induce apoptosis. Early apoptotic cells, which are in the beginning of apoptosis, are distinguished from already dead cells (late apoptosis). The data indicated that treatment with N6L in various concentrations for 24 h has no effect on the survival of HUVECs and confirmed that N6L has a direct effect on endothelial cells adhesion and migration and secondarily on their proliferation.

### N6L down-regulates MMP-2 in HUVEC

**Figure 3:**
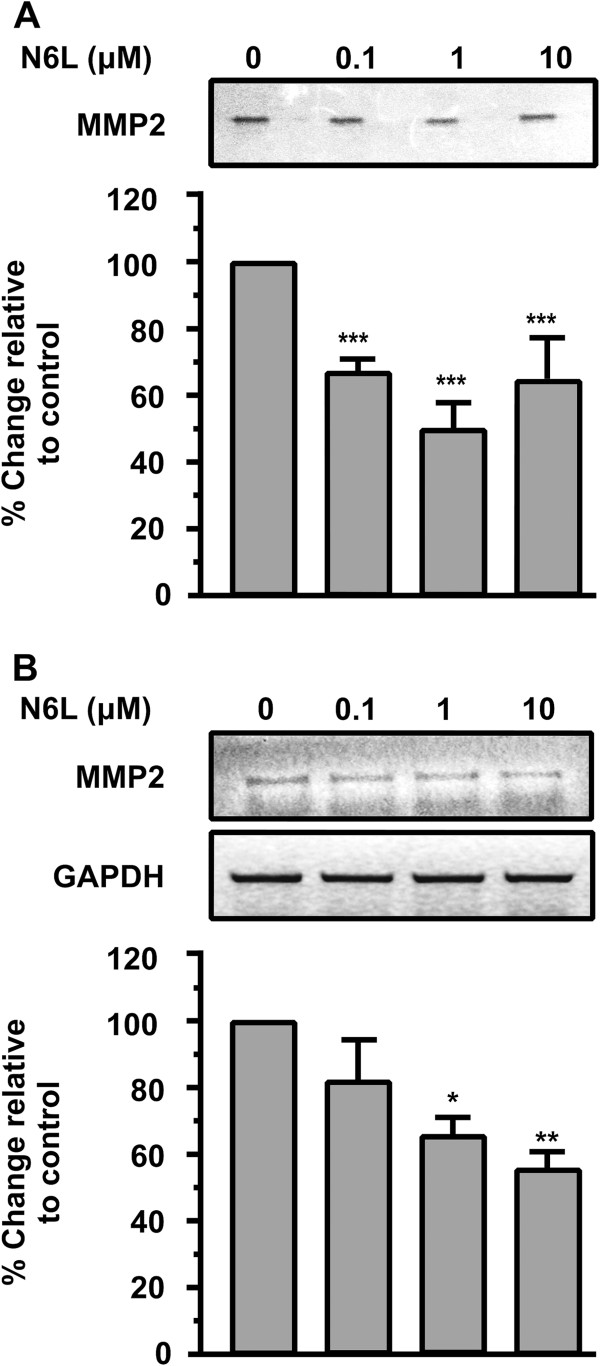
**N6L down-regulates MMP-2 in HUVECs. (A)** Endothelial cells were cultured in a minimal medium with increasing concentrations of N6L and 8 h later the supernatants were analyzed for MMP-2 activity by zymography. Results are expressed as % change relative to control and are mean values ± SE from at least 3 independent experiments. **(B)** Endothelial cells were incubated with increasing concentrations of N6L and 24 h later total RNA was extracted from the cells, RT-PCR reactions were performed using specific primers for MMP-2 or GAPDH mRNAs, the PCR products were analyzed in agarose gels and quantified. Results are expressed as % change relative to control and are mean values ± SE from at least 3 independent experiments.

We next studied the effect of N6L on the activation of Matrix Metalloproteinases (MMPs) which are involved in the degradation of extracellular matrix and required for cell migration. MMPs play a crucial role in angiogenesis, as they digest the ECM and facilitate the cell’s migration. MMP-2 is expressed in endothelial cells as well as in most of the cell types and is important for endothelial cell migration and vascular remodelling during angiogenesis [21,22]. It also facilitates the migration of tumor cells [23]. The results showed that MMP-2 activity was markedly reduced by 1 μM of N6L (Figure 3A). We further demonstrated that N6L suppressed the expression of MMP-2 mRNA determined by RT-PCR (Figure 3B). The results indicated that both enzyme activity and expression of MMP-2 was inhibited by N6L.

The results suggested that N6L and therefore nucleolin might affect the expression of genes involved in proteolytic activation.

### Inhibition of SRC, FAK, AKT and ERK1/2 activation during N6L stimulation of HUVECs in vitro

Several studies have indicated that the SRC, FAK, AKT and MAPK members are involved in the signaling cascade that regulates angiogenesis [24,25]. Consequently, the effects of N6L on the phosphorylation status of these molecules in HUVECs were investigated.

**Figure 4:**
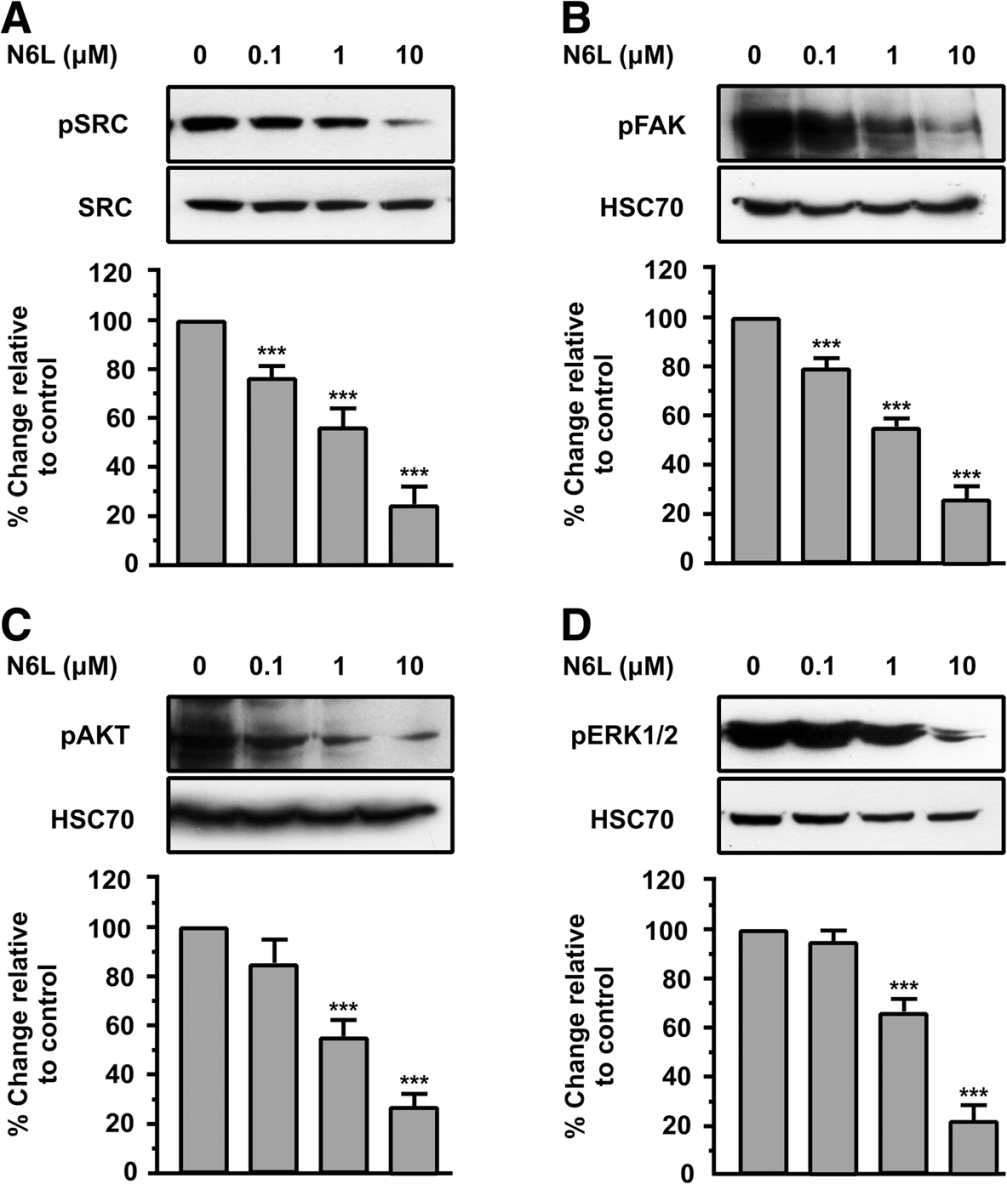
**N6L signaling down-regulates pSRC, pFAK, pAKT and pERK1/2.** Western blot analysis of phosphorylated SRC, FAK, AKT and ERK1/2, in cells stimulated by increasing concentrations of N6L for 15 minutes. The blots were stripped and re probed for total SRC or HSC70. Results are expressed as % change relative to the control and are mean values ± SE from at least 3 independent experiments.

As shown in Figure 4A, N6L promoted a decrease in SRC phosphorylation within 15 min in a concentration dependent manner with a maximal effect (80% inhibition relative to control) at a concentration of 10 μΜ. Similarly, we found that FAK, AKT and ERK1/2 were also inactivated after a15-min incubation with N6L in a concentration dependent manner with a maximal inhibition of more than 75% as compared to the control.

Taken together, these data indicated that N6L inhibits the phosphorylation of all of these kinases in a dose-dependent manner (Figure 4A, B, C and D). Results showed that the anti-angiogenic action of N6L might partly occur through a suppression of SRC, FAK, AKT and ERK pathways, but further research is needed in order to understand the mechanism of action of N6L and how it inhibits the activation of these signaling pathways.

### Down regulation of Nucleolin expression inhibits adhesion and proliferation of HUVECs and blocks the inhibitory action of N6L

Previous reports have shown that N6L interacts specifically with cell surface nucleolin [20,26]. N6L has been reported to also bind nucleophosmin on the cell surface but with considerably lower affinity than nucleolin [20,26].

In order to confirm that N6L exerts the previously described biological actions mainly through nucleolin, as well as the involvement of cell-surface nucleolin in these effects, we transiently transfected HUVECs with a siRNA targeting the mRNA of nucleolin. In parallel, HUVECs were transiently transfected with a siRNA that does not target any mRNA (negative control) (data not shown).

**Figure 5:**
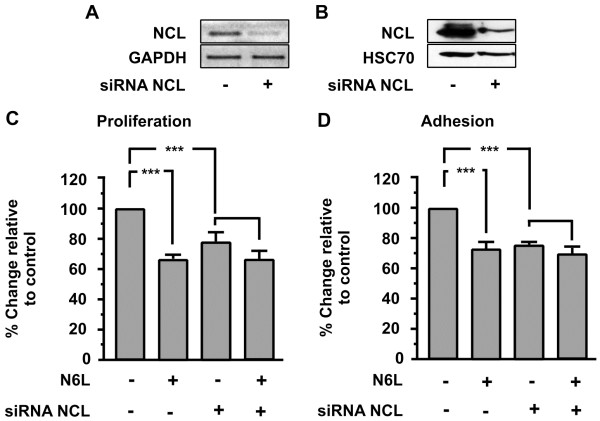
**Effect of nucleolin (NCL) knockdown on N6L biological actions.** Down-regulation of nucleolin (NCL) mRNA **(A)** and protein **(B)** using specific siRNA targeting nucleolin mRNA. Effect of N6L (10μΜ) on proliferation **(C)** and adhesion **(D)** of HUVECs. The last two bars of each diagram indicate HUVECs that were transiently transfected with siRNA targeting nucleolin (NCL). Results are expressed as % change relative to control and are mean values ± SE from at least 3 independent experiments.

The analysis of the expression of NCL indicates that using specific siRNA targeting nucleolin mRNA results in a reduction in the levels of mRNA and protein up to 80% respectively (Figure 5A and B).

As shown in (Figure 5C and D), NCL knockdown blocked the inhibitory effect of N6L in HUVECs proliferation and adhesion respectively. Moreover, the same experiment shows that nucleolin is a crucial factor in the proliferation and adhesion of HUVECs since the down-regulation of nucleolin expression by itself results in the inhibition of proliferation and adhesion.

### Down regulation of Nucleolin expression inhibits SRC and ERK1/2 activation in HUVECs and blocks the inhibitory action of N6L

In this study, we show that N6L inhibits phosphorylation of several kinases implicated in angiogenesis pathways in many ways.

**Figure 6:**
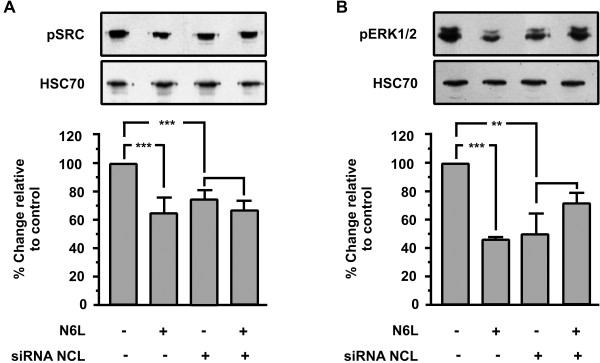
**Effect of nucleolin (NCL) knockdown on N6L-induced signal transduction.** Western blot analysis of phosphorylated SRC and ERK1/2 in HUVECs stimulated with 10 μM N6L for 15 minutes. The blots were stripped and re probed for HSC70. The last two bars of each diagram indicate HUVECs that were transiently transfected with siRNA targeting nucleolin. Results are expressed as % change relative to control and are mean values ± SE from at least 3 independent experiments.

To confirm that N6L signaling takes place mainly through nucleolin interaction, we tested the N6L effect on activation of SRC and ERK1/2 of transiently transfected HUVECs with siRNA for nucleolin mRNA. As shown in (Figure 6A and B), the phosphorylation levels of SRC and ERK1/2 are induced on transiently transfected HUVECs compared with wild type cells, while nucleolin knockdown blocked N6L-induced SRC and ERK1/2 inactivation.

Taken together, these results indicate that N6L/nucleolin interaction affects various signal transduction pathways that reduce the phosphorylation levels of these signal transduction molecules, and are involved in the inhibition of cellular adhesion and proliferation.

## Discussion

Cell-surface nucleolin has been described by several studies as a molecule that is involved in the growth of tumor cells as well as in angiogenesis [3,20,26,27]. Effective inhibition of tumor cell proliferation and impairment of angiogenesis have been succeeded by targeting surface nucleoproteins, like nucleolin, with N6L and HB-19 [20,26,27]. Therefore, functional blockade of surface nucleoproteins seems to result in a general inhibitory mechanism that is not specific to a single pathway related to one growth factor implicated in carcinogenesis.

In previous studies, our group has shown the dual action of N6L and HB-19 on tumor and endothelial cells pointing out surface nucleoproteins as an important anticancer therapy target [20,27].

N6L, which like HB-19, binds nucleolin at the RGG domain of its C-terminal end, was synthesized aiming the improvement of HB-19 biological actions. N6L presents hexavalently the pseudo-tripeptide Lysψ(CH_2_N)-Pro-Arg while HB-19 presents it pentavalently. The inhibitory action of N6L regarding HIV-1 entry is 4-fold compared to that of HB-19 and the down-regulation of surface/cytoplasmic nucleolin is greater in cells treated with N6L compared to cells treated with HB-19 [26]. Furthermore, N6L showed greater inhibitory action in tumor cells growth compared to HB-19 [20]. N6L, but not HB-19, has been reported to also bind nucleophosmin on the cell surface but with considerably lower affinity than nucleolin [20,26].

In this study, we sought to investigate the biological actions of N6L in HUVECs. We found that N6L inhibits in vitro HUVEC cell adhesion, migration and motility in a concentration dependent manner (Figure 1A, C and D). If left for more than 6 h, HUVECs will adhere and migrate even in the presence of N6L(data not shown), therefore we investigated the effect of N6L in proliferation of HUVECs showing that it is also inhibited, even if it is a secondary effect (Figure 1B). The inhibitory effect of N6L on angiogenesis [20], as well as on HUVECs migration and motility may be associated with down regulation of MMP-2. HB-19, which is similar to N6L, was shown to down regulate MMP-2 expression [28,29]. We found that both enzyme activity and expression of MMP-2 were inhibited by N6L (Figure 3). Furthermore, we found that N6L treatment shows no toxicity in HUVECs in vitro, as N6L didn’t induce apoptosis (Figure 2). N6L induces apoptosis of tumor cells in vitro and reduces tumor growth in mice without evidence of toxicity [20]. N6L and other Nucants are reported to exert distinct inhibitory mechanisms depending on the malignant tumor cell type indicating that the capacity of Nucant to induce cell death is by a selective mechanism [20,26]. The lack of translocation of N6L to the nucleus probably accounts for its lack of toxicity [20,26]. The mechanism through which N6L induces apoptosis in some tumor cells without affecting cell viability in other tumor cells and normal cells, like HUVECs, remains to be investigated.

Ligant binding to surface nucleolin could generate intracellular Ca2+ membrane fluxes and initiate signal transduction events [30]. HB-19 binds the C-terminal RGG domain of cell-surface expressed nucleolin and blocks its function as a receptor or binding molecule for various ligands [31,32]. N6L also binds the C-terminal RGG domain of cell-surface expressed nucleolin and therefore it might exert its biological action in a similar way. In order to identify the signaling pathways that N6L affects and through which exerts its biological action, we investigated the effect of this peptide on well known angiogenic signaling pathways mediated by many of these ligands of nucleolin. We found that N6L inhibits the phosphorylation levels of SRC, FAK, AKT, and ERK1/2 in a concentration-dependent manner showing that the anti-angiogenic action of N6L might partly occur through suppressing SRC, FAK, AKT and ERK pathways.

Down regulating nucleolin, we found that the proliferation and adhesion of HUVECs was inhibited, highlighting the importance of nucleolin in these biological actions (Figure 5C and D). To confirm that our siRNA was specific for NCL we examined the effect of siRNA on the expression of GAPDH mRNA as shown in Figure 5A and other molecules such as tubulin (data not shown).

The inhibitory effect of N6L on cellular proliferation and adhesion was almost completely blocked in the transfected HUVECs compared to the wild type (Figure 5C and D last two columns). Furthermore, the phosphorylation levels of SRC and ERK1/2 are induced on transiently transfected HUVECs compared with wild type cells (Figure 6, first and third columns), while nucleolin knockdown blocked N6L-induced SRC and ERK1/2 inactivation (Figure 6 last two columns). In Figure 5B the reduction of HSC70 levels in siRNA transfected cells does not indicate secondary inhibitory effects of our siRNA on the expression of cellular proteins. We used HSC70 for western blot normalization and its reduced levels are a consequence of the reduced number of cells due to the inhibitory effect of NCL knock-down on cell proliferation. Taken together, these results indicate that the complex N6L/nucleolin interaction triggers a signal transduction pathway that reduces the phosphorylation levels of these signal transduction molecules, and also that it inhibits cellular adhesion and proliferation. Down regulation of mRNA of NCL using siRNA is reported to reduce surface nucleolin [7]. Additionally, the expression of nucleolin on the surface of endothelial cells is due to the constant induction of nucleolin mRNA, as NCL mRNA and cell-surface-NCL have half-life time of about 45-90 min, whereas nuclear NCL has more than 24 h [6]. Therefore, although we did not distinguish surface and nuclear nucleolin, we can presume with relative safety, that cell surface NCL levels were reduced in our experimental conditions. As a result, we can hypothesize the involvement of surface NCL in N6L action and consider NCL the main molecule through which N6L exerts its action, knowing that there is more research to be done in order to extrapolate that the mechanism by which N6L exerts its action is through surface NCL.

## Conclusions

In summary, in this study we shed light on the role of cell surface nucleoproteins in the regulation of their binding proteins signaling and activity in HUVECs. These results indicate that N6L could constitute an interesting tool to inhibit angiogenesis. This possibility is reinforced by the fact that these multimeric pseudopeptides are synthetic molecules which are stable in serum, lack tissular toxicity and whose production can easily be up scaled, providing novel therapeutic opportunities in proliferative diseases.

## Methods

### Endothelial cell culture

Human umbilical vein endothelial cells (HUVEC) were isolated from umbilical cord vein by collagenase digestion as previously described [33] and used at passages 2–4. The cells were grown as monolayers in medium M199 supplemented with 15% fetal bovine serum (FBS), 150 μg/ml of endothelial cell growth supplement, 5 U/ml heparin sodium, 100 U/ml penicillin-streptomycin and 50 μg/ml gentamycin. Cultures were maintained at 37°C, 5% CO_2 _and 100% humidity.

## Materials

Cell culture reagents were from BiochromKG (Seromed, Germany). All other reagents were purchased from Sigma-Aldrich. Monoclonal antibodies against pSRC-kinase (Tyr416), pFAK (Tyr925), pAKT (Ser473), pERK^1^/_2 _(Thr202/Tyr204) and total SRC were purchased from Cell Signaling Technology. Polyclonal antibodies against HSC70 and NCL (nucleolin) were purchased from Santa Cruz Biotechnology, Inc.

### Peptide construct

N6L was synthesized, as previously described, using the solid phase peptide methodology [20].

### Cell proliferation assay

An equal number of cultured HUVECs in medium containing 15% FBS were left to adhere for 20 h in a cell culture microplate. Then, they were treated with different N6L concentrations and were allowed to proliferate for 3 days. The cell number was estimated by the crystal violet assay. Data are the mean ± SEM of at least three independent experiments.

### Adhesion assay

24-well culture plates were coated with 1% v/v gelatin for 20 min at 37°C. 50,000 resuspended cells incubated in M199 medium with different concentrations of N6L for 30 min and then seeded. After a 30 min incubation period unattached cells were removed by shaking the plates at 2,000 rpm for 10 sec, and by three washes with PBS. Attached cells were fixed with methanol and stained with crystal violet.

### Boyden chamber assay

Migration assays were performed in a 24-well microchemotaxis chamber (Costar, Avon, France), using untreated polycarbonate membranes with 8 μm pores. HUVECs were harvested and resuspended at a concentration of 10^5 ^cells/0.1 ml in the corresponding medium containing 0.25% bovine serum albumin (BSA). The bottom chamber was filled with 0.6 ml of the corresponding medium containing 0.25% BSA and N6L. The upper chamber was loaded with 10^5 ^cells and incubated for 4 h at 37°C. After completion of the incubation, filters were fixed. Non-migrated cells were scrapped off the upper side of the filter, and filters were stained with crystal violet. The number of migrated cells was quantified by counting the entire area of the filter using a grid and an Optech microscope at a 20× magnification.

### In vitro endothelial cell wound healing assay

HUVECs were cultured in 6-well plates at 2×10^5 ^cells/well as confluent monolayers. The monolayers were wounded in a line across the well with a 200 μl standard pipette tip. The wounded monolayers were then washed twice with PBS to remove cell debris and were incubated with increasing concentrations of N6L for 48 h. The area of the initial wound was photographed using a charge-coupled device camera connected to an inverted microscope (Axiovert 35; Zeiss, Thornwood, NY). The wound healing effect was calculated in comparison with the area of the initial wound.

### Western blot analysis

Cells were starved for 4 h and then incubated with different concentrations of N6L for 15 min. Cells were subsequently washed twice with PBS and lysed in 250 μl 2X SDS loading buffer under reducing conditions. Proteins were separated by SDS-PAGE and transferred to an Immobilon-P membrane for 3 h in 48 mM Tris pH 8.3, 39 mM glycine, 0.037% SDS, and 20% methanol. The membrane was blocked in TBS containing 5% non-fat milk and 0.1% Tween 20 for 1 h at 37°C. Membranes were then probed with primary antibody overnight at 4°C under continuous agitation. All antibodies were used at a 1:1000 dilution. The blot was then incubated with the appropriate secondary antibody coupled to horseradish peroxidase, and bands were detected with the ChemiLucent Detection System Kit (Chemicon International Inc., CA) according to the manufacturer’s instructions. Where indicated, blots were stripped in buffer containing 0.5 mM Tris HCl pH 6.8, 2% SDS, 100 mM 2-mercaptoethanol for 30 min at 56°C and re probed. A quantitative estimation of band size and intensity was performed through analysis of digital images using the ImagePC image analysis software (Scion Corporation, Frederick, MD).

### Gelatin zymography

Secreted metalloproteinases were detected and characterized by zymography. Conditioned media were obtained by an 8 h incubation of cells in serum-free media. Conditioned media were loaded onto 10% SDS-PAGE gels that had been co polymerized with 1 mg/ml gelatin. Electrophoresis was carried out under non-reducing conditions at 100 V for 2 h at 4°C. Gels were washed once for 60 min in 2.5% Triton X-100 to remove SDS and incubated in zymogen activator buffer (50 mM Tris–HCl pH 7.6, 10 mM CaCl_2_, 0.2 M NaCl) for 24 h at 37°C. Gels were stained with 0.5% Coomassie blue in 30% methanol/10% acetic acid for 30 min at room temperature and de-stained in 30% methanol/10% acetic acid three times for 15 min. The presence of metalloproteinases was indicated by an unstained (due to proteolysis) zone in the substrate. Both active forms and pro-enzymes are revealed by this technique, since exposure of pro-MMPs to SDS during SDS-PAGE leads to activation without proteolytic cleavage. The relative amounts of MMPs were quantified by NIH Image Analysis software. The normalization was based on the number of cells of each well (using crystal violet method).

### Annexin V binding staining

The analysis of annexinV binding was carried out with an Annexin V-FITC Detection Kit I (PharMingen, San Diego, CA) according to the manufacturer’s instructions. An equal number of cultured HUVECs in medium containing 15% FBS were left to adhere for 20 h in a cell culture microplate. They were then treated with different N6L concentrations and collected after 24 h, washed twice with cold PBS, centrifuged at 200 g for 5 min and resuspended in binding buffer at a concentration of 10^6 ^cells per ml. 100 μl of the solution were transferred to a 5 ml culture tube and 5 μl of annexin V-FITC and 5 μl of PI were added. Cells were gently vortexed and incubated for 15 min at room temperature in the dark. Finally, 400 μl of binding buffer was added to each tube, and samples were analyzed by FACScan flow cytometer (Becton Dickinson). For each sample, 10,000 un gated events were acquired. PI (-)/annexin (+) cells represent early apoptotic populations and PI (+)/annexin (+) cells represent late apoptotic populations.

### Crystal violet assay

Adherent cells were fixed with methanol and stained with 0.5% crystal violet in 20% methanol for 20 min. After gentle rinsing with water, the retained dye was extracted with 30% acetic acid, and the absorbance was measured at 595 nm.

### Reverse transcriptase-polymerase chain reaction (RT-PCR) for Nucleolin, MMP2, and GAPDH

Total RNA was extracted using the Nucleospin RNA II kit (Macherey-Nagel, Germany) according to the manufacturer’s instructions. The integrity of isolated RNA was examined by electrophoresis on a 1% agarose gel containing 0.5 mg/ml ethidium bromide. Specific primers were as follows:

Nucleolin,

5′′- TGCCAAGAAGACAGTTACACCA -3′′ and

5′′- AGGAACAACTTTTGCAGCTTTC - 3′′;

MMP2,

5′′- ACAGTCCGCCAAATGAACC - 3′′ and

5′′- CCTGGGCAACAAATATGAGA -3′′

GAPDH,

5′′-CCACCCATGGCAAATTCCATGGCA-3′′ and

5′′ TCTAGACGGCAGGTCAGGTCCACC-3′′.

The RT-PCR reactions were performed in a single step with 250 ng of total RNA, using the Qiagen RT-PCR system. The RT-PCR products were subjected to electrophoresis on 1% agarose gel containing 0.5 mg/ml ethidium bromide, digitally photographed, and quantified using image analysis software (Scion Image PC, Scion Corporation, Frederick, MD).

### siRNA transfection

RNA oligonucleotide primers were obtained from Ambion Inc and the Lipofectamine RNAiMAX Transfection Agent was obtained from Invitrogen. The following sequences were used:

siRNA Nucleolin sense: 5′′-GGAUAGUUACUGACCGGGA-3′′;

siRNA Nucleolin antisense: 5′′-UCCCGGUCAGUAACUAUCC-3′′,

HUVECs were plated in 6 wells-plates and incubated for 24 hours at 37°C. Cells were then transfected at a final concentration of 10nM siRNA using Lipofectamine RNAiMAX reagent (Invitrogen) according to the manufacturer’s instructions.

Transfection efficiency was evaluated using Silencer FAM Labelled GAPDH siRNA (Ambion). Negative control siRNAs from Ambion was also used.

### Statistical analysis

Comparisons of the mean values among groups were performed by means of ANOVA and unpaired Student t-test. Homogeneity of variances was tested by Levene’s test. Each experiment included at least triplicate measurements for each condition tested. All results are expressed as mean ± SE from at least three independent experiments. Values of p less than 0.05 were accepted as significant (*p < 0.05, **p < 0.01, ***p < 0.001).

## Competing interests

All authors have declared that they have no competing interest.

## Authors’ contributions

All authors participated in the design of the study. BC performed all experiments, performed the statistical analysis and drafted the manuscript. BJP constructed and provided the peptides. CJ helped to draft the manuscript. KP conceived of the study, participated in its design and coordination and helped to draft the manuscript. All authors read and approved the final manuscript.
